# Supplementary material for: Interfacial ice sprouting during salty water droplet freezing
Source: Nat Commun. 2024 Mar 13;15:2249. doi: 10.1038/s41467-024-46518-y (PMC10937636; doi:10.1038/s41467-024-46518-y)
Supplement: Supplementary file 1 — Supplementary Information [file 41467_2024_46518_MOESM1_ESM.pdf]

Supplementary Information for

Interfacial ice sprouting during salty water droplet freezing

Fuqiang Chu[1,2], Shuxin Li[1], Canjun Zhao[3], Yanhui Feng[1,2], Yukai Lin[3], Xiaomin Wu[3*],*

Xiao Yan[4,5,6], Nenad Miljkovic[6,7,8,9*]*

[1]School of Energy and Environmental Engineering, University of Science and Technology Beijing, Beijing 100083, China

[2]Beijing Key Laboratory of Energy Conservation and Emission Reduction for Metallurgical Industry, University of Science and Technology Beijing, Beijing, 100083, China

[3]Department of Energy and Power Engineering, Tsinghua University, Beijing 100084, China

[4]Key Laboratory of Low-grade Energy Utilization Technologies and Systems, Chongqing University, Ministry of Education, Chongqing 400030, China

[5]Institute of Engineering Thermophysics, Chongqing University, Chongqing 400030, China

[6]Department of Mechanical Science and Engineering, University of Illinois at Urbana–Champaign, Urbana, IL 61801, USA

[7]Department of Electrical and Computer Engineering, University of Illinois at Urbana–Champaign, Urbana, IL 61801, USA

[8]Materials Research Laboratory, University of Illinois at Urbana–Champaign, Urbana, IL 61801, USA

[9]International Institute for Carbon Neutral Energy Research (WPI-I2CNER), Kyushu University, 744 Moto-oka, Nishi-ku, Fukuoka 819-0395, Japan

***Corresponding Authors:** yhfeng@me.ustb.edu.cn
wuxiaomin@mail.tsinghua.edu.cn
yanx23@cqu.edu.cn
nmiljkov@illinois.edu

Table of Contents:

Supplementary Sections

S1 Experimental surfaces

S2 Experimental setup for droplet icing

S3 Temperature curves inside droplets and on droplet surfaces during icing

S4 Determination of inflection points on the temperature curves

S5 Effect of location of thermocouples on the temperature curve and its inflection point

S6 Molecular dynamic simulation of salty water icing

S7 Growth of dense ice crystals during the freezing stage of salty droplets

S8 Semi-empirical model to predict the freezing time of salty droplets

S9 Estimation of the brine film thickness on top of freezing salty droplets

S10 Analogy experiments for the measurement of ice precipitation rate and condensation rate

S11 Condensation phase change driving force

S12 Effects of air humidity and surface temperature on the ice sprouting phenomenon

S13 Effect of salt ions on the ice sprouting phenomenon

S14 Salty droplet icing on hydrophilic surfaces

Supplementary References

S1 Experimental surfaces

In this work, we prepare Al-based experimental surfaces for icing experiments, which are made by the chemical etching–deposition method. We construct micro and nano structures on a metal aluminum substrate through displacement reaction, and then modify the structured surface with low surface energy substances to obtain the surface. The first step is the cleaning of the metal surfaces. The Al foils are first polished with the fine sand paper to remove the surface oxidation layer and then immerse into the absolute ethyl alcohol and deionized water for the ultrasonic cleaning in proper order. The second step is the chemical processing of the metal surfaces. The cleaned Al foils are immersed into acid-salt mixed solution (0.1 mol/L HCl and 0.1 mol/L $CuSO_4$) for 5-10 min. Gas bubbling and solid deposition can be observed on the metal surfaces during this step. The third step is the modification of the metal surfaces. The processed metal foils are immersed into the alcohol solution of 1 wt.% fluoroalkyl silane (1H,1H,2H,2H-Perfluorodecyltriethoxysilane) for 30 min and then dried in a drying oven at 100°C for an hour.

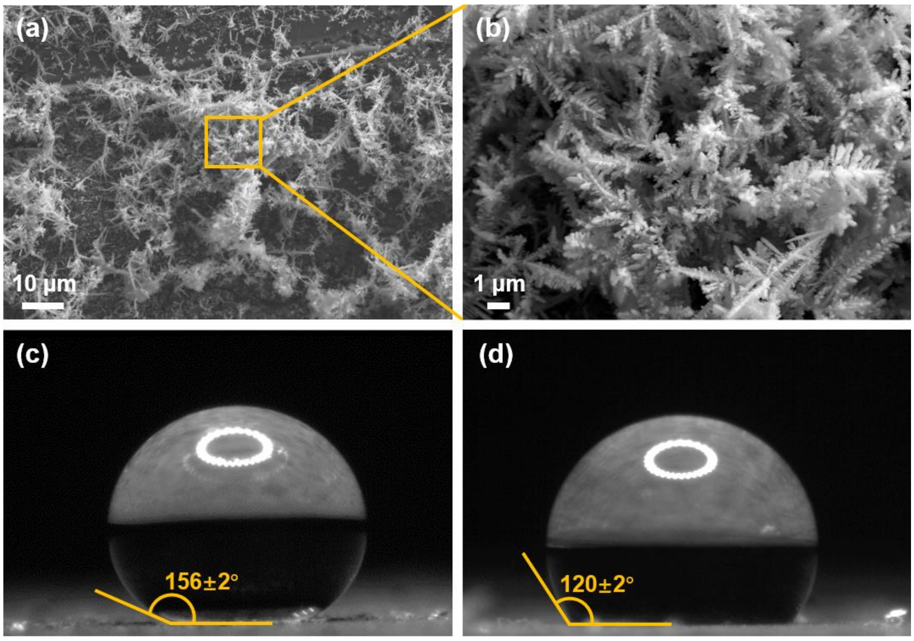

FIGURE S1. Characterization of experimental surfaces. (a) and (b) SEM images of the experimental surface. (c) and (d) Contact angle measurement of the experimental surface under environmental temperature (25°C) and supercooled temperature (−15°C). The standard deviations are based on five measurements.

As the SEM images shown in Figure S1a, the surface has hierarchical micro and nano scale structures, which are like moss growing on the substrate surface with ravines that spread in all directions. The enlargement in Figure S1b shows that the moss-like structures are composed of smaller stems and leaves which are less than 1 μm in size. At environmental temperature, the experimental surface is superhydrophobic with a contact angle of 156±2°, but when the surface is −15°C under supercooled conditions, its superhydrophobicity is lost and the contact angle decreases to 120±2°, as shown in Figures S1c and S1d. This is mainly due to the capillary condensation inside the micro structures and thin water film formation adjacent to the triple phase line[1,2]. The contact angle decreasing also indicates a wetting transition from Cassie-Baxter state to Wenzel state[3]. Therefore, under our experimental conditions for droplet icing, the experimental surface is hydrophobic, which facilitates the observation and shooting of the icing process.

S2 Experimental setup for droplet icing

Figure S2a shows the schematic of experimental setup for droplet icing experiments. The experimental setup mainly consists of a semiconductor thermoelectric refrigeration module, a temperature & humidity acquisition module, and a high-speed microphotography module. The semiconductor thermoelectric refrigeration module is used for providing cold sources for icing and is composed of a semiconductor cooler, a DC power supply, a water cooler, and a constant temperature bath circulator. The temperature and humidity acquisition module is used for monitoring icing condition changes and consists of T-type thermocouples (Omega TT-T-30, 0.254 mm cable diameter, ±0.5ºC accuracy), a temperature-humidity sensor (Testo 400, ±0.5ºC accuracy for temperature and ±3% accuracy for humidity), a data acquisition, and an infrared camera (FLIR A615, USA). The high-speed microphotography module mainly consists of a top-view microscope (AOSVI 3M150G, China), a side-view high-speed optical camera (Photron FASTCAM Mini UX100, Japan), a light source, and a high-performance computer. In addition to the icing experiments of real three-dimensional droplets, we also design a Hele-Shaw cell based on literature[4] to observe icing of a droplet slice. As the schematic and photo in Figure S2b show, the Hele-Shaw cell is composed of two glass sidewalls and two fixtures for glass walls.

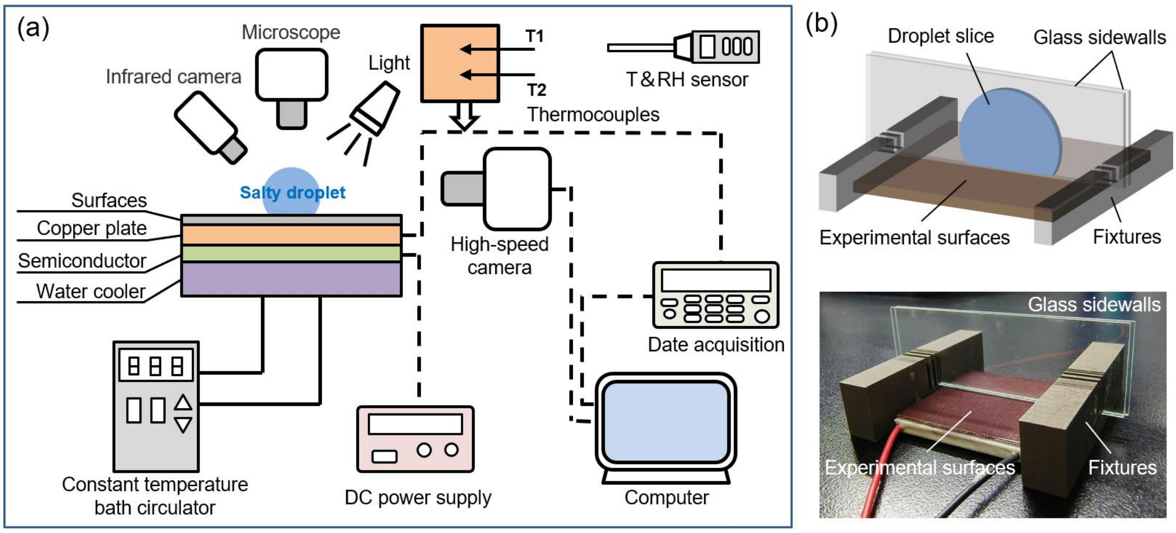

FIGURE S2. Experimental setup. (a) Schematic of the entire experimental system. Schematic not to scale. The system mainly consists of a semiconductor thermoelectric refrigeration module, a temperature and humidity acquisition module, and a high-speed microphotography module. (b) Schematic and photo of the Hele-Shaw cell used in our experiments.

S3 Temperature curves inside droplets and on droplet surfaces during icing

In this work, we need to measure the temperature variation of the icing droplet. As shown in Figure S3a, when measuring the droplet temperature, the thermocouples are located in the middle of the droplet. In the temperature curves during icing, the nucleation/recalescence stage and the freezing stage are clearly distinguished in both water droplet and salty droplet. Especially, the nucleation/recalescence stage of salty droplet icing lasts one order of magnitude longer than that of pure water droplet, and its freezing stage also has a longer duration. The circle marks the inflection point in the curve, which is just the end of the freezing stage. However, it should be noted that for salty droplet icing and pure water droplet icing, there is a difference in the physics behind the inflection point. For salty droplet icing, the inflection point means the slope of the temperature curve becomes zero; while for pure water droplet icing, the inflection point indicates a change in the slope of the temperature curve (Figure S3a). This is because a pure water droplet undergoes a solid cooling stage after the freezing stage ends. The term solid cooling refers to the temperature of the solid ice droplet continuing to decrease because of the colder surface (see red line in Figure S3a). There is no instant solid cooling stage for salty droplet icing, an important difference between salty droplet icing and pure water droplet icing.

In order to obtain the temperature curve at the top and bottom edge of a salty droplet icing, we use an infrared camera to record temperature variation, as shown in Figure S3b, the circle marks the end of the freezing stage. From the infrared temperature data, the top temperature of the droplet does not change after the freezing ends, indicating that the brine film on top of the droplet is saturated at its temperature.

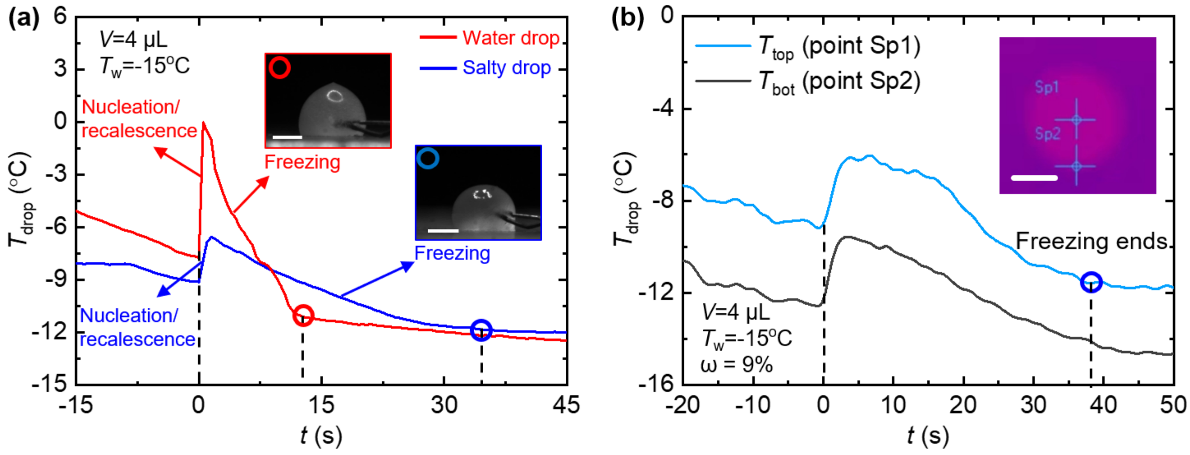

FIGURE S3. Temperature measurement of icing droplets. (a) Temperature curves during icing measured by thermocouples in the middle of a water droplet (V= 4 μL and T_w= −15°C) and a salty droplet (V= 4 μL, ω=9% and T_w= −15°C). The circle marks the inflection point in the curve, which is just the end of the freezing stage. The inset beside it shows the droplet snapshot at the corresponding moment. (b) Temperature curves during icing measured by the infrared camera at the top and bottom edge of a salty droplet (V= 4 μL, ω=9% and T_w= −15°C). The circle marks the end of the freezing stage. All scale bars indicate 1 mm.

S4 Determination of inflection points on the temperature curves

The inflection point on the temperature curve is quantitatively determined by taking the derivative of the temperature curve. Figures 2A-B and related contents have shown the procedure of the inflection point determination. In Figure S4, we show another two sets of data under the same icing conditions (V=4 μL, ω=9% and T_w=-15°C) as those in Figures 2A-B, the left side of the figure shows two temperature curves, and the right side shows the derivatives of the temperature curves in the dashed boxes. The threshold of derivative of the temperature curve is set to be -0.01. When the derivatives of the curves rise from a large negative value to -0.01, the inflection points (marked by red circle) are reached. Within the allowable range of error, the times of inflection points on the three temperature curves in Figure 2A and Figure S4 are consistent, indicating the reliability of the determination method for the inflection point.

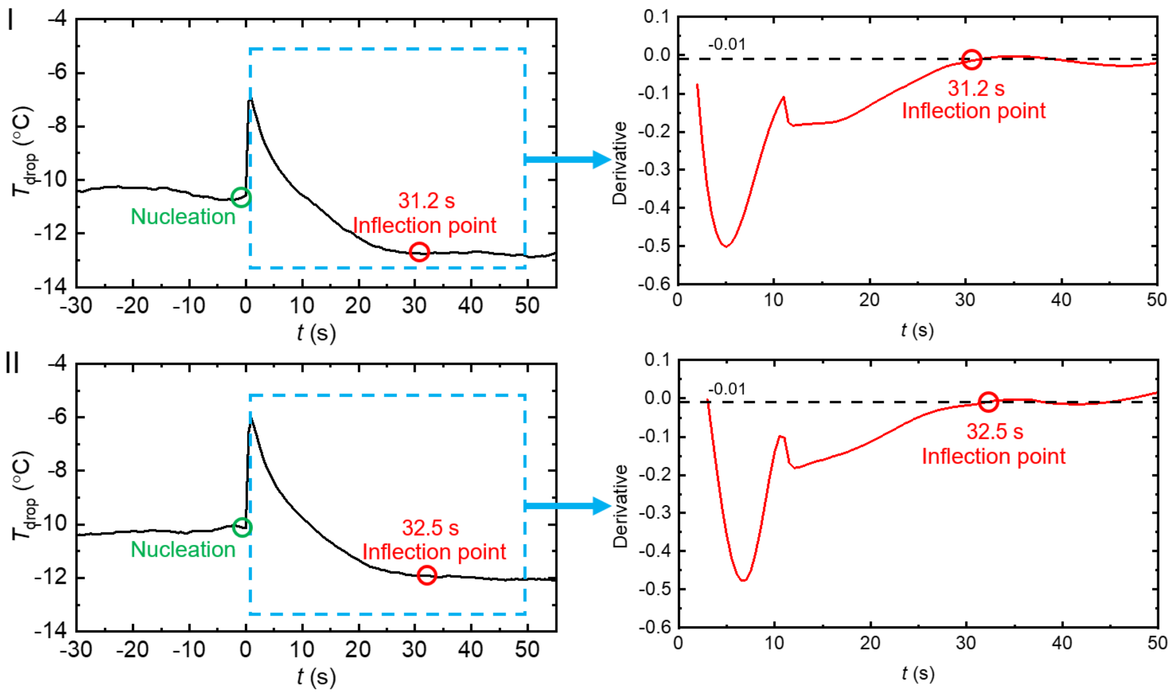

FIGURE S4. Determination of the inflection point on the temperature curves during icing of salty droplets. Two sets of data under the same icing conditions (V= 4 μL, ω=9% and T_w= −15°C) are shown here, the left side of the figure shows two temperature curves, and the right side shows the derivatives of the temperature curves in the dashed boxes. Notice that the temperature curves always have some fluctuations, so we smooth the derivative curves using Savitzky-Golay method.

S5 Effect of location of thermocouples on the temperature curve and its inflection point

Since a temperature gradient exists in the ice layer inside a freezing droplet, to avoid the influence of location of thermocouples on the measured temperature curve and the inflection point, we place two thermocouples at different locations inside a salty droplet (Figure S5a) and measure the temperature variations during the droplet icing. As shown in Figure S5b, at a certain moment, the temperatures of two thermocouples rapidly rise simultaneously, and this moment just represents the onset of nucleation (defined as zero moment here). This result is reasonable, because the nucleation is a rapid kinetically controlled process[5,6], during which, the supercooling inside the droplet disappears, and the overall temperature of the droplet rises. Therefore, the onset of nucleation can be determined based on the temperature change inside the droplets.

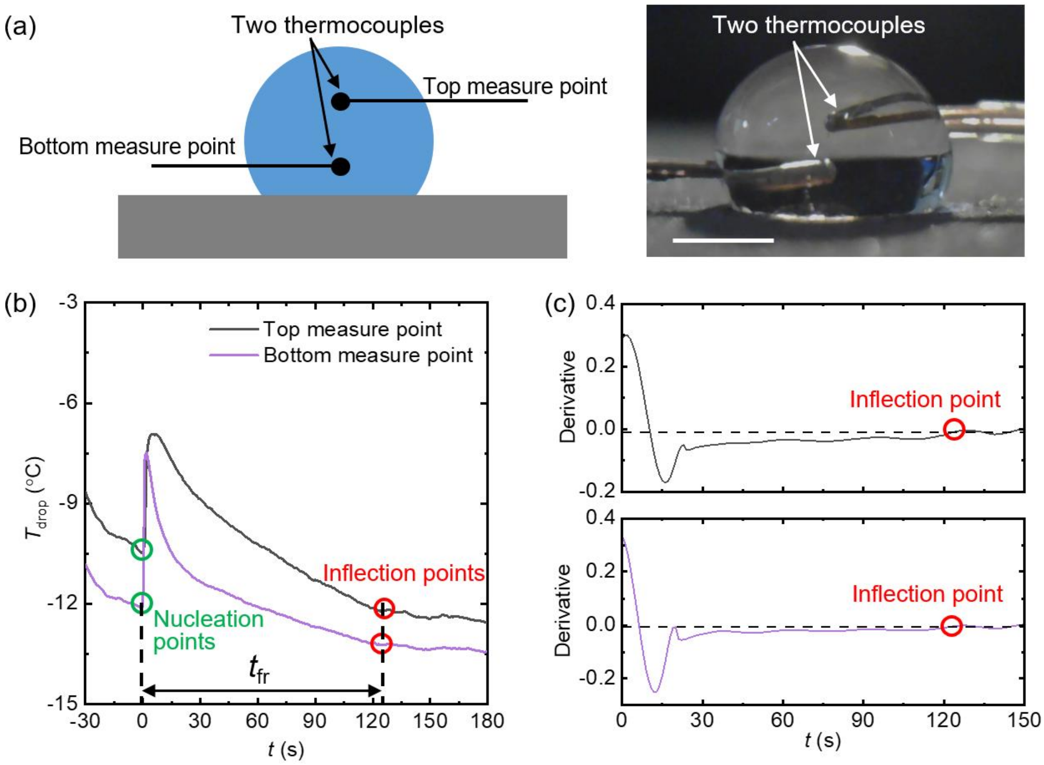

FIGURE S5. Temperature measurement by two thermocouples at different locations inside a salty droplet (V=40 μL, ω=9% and T_w=-15°C). (a) Schematic and experimental photo of locations of two thermocouples inside the salty droplet. The scale bar is 2 mm. (b) Temperature curves measured by two inserted thermocouples during the salty droplet icing. (c) Derivatives of the two temperature curves (partial) for the determination of the inflection points. Although there is a temperature gradient in the ice layer inside the droplet, both the onset of nucleation (marked by green circles) and the time of

inflection point (marked by red circles) on the two temperature curves are consistent. It should be noted that to insert two thermocouples, we use a larger droplet here.

The freezing time of a salty droplet is defined as the duration from the nucleation to the inflection point in the temperature curve. As mentioned above, the nucleation onset in the temperature curve is the moment occurring a sudden temperature rise. As for the inflection point in the temperature curve, we quantitatively determine it by taking the derivative of the temperature curve (as shown in Figure 2A-B and Figure S4). Based on this rule, we respectively determine two inflection points on the two temperature curves inside the salty droplet, as marked by red circles in Figure S5b and S5c. As shown, the moments of the two inflection points on two temperature curves are consistent, indicating that every temperature curve inside the droplet (such as that in Figure 2A) can be used to determine the end of the droplet freezing stage, as well as the freezing time. In other words, the effect of location of thermocouples on the measured temperature curve and the inflection point is eliminated.

S6 Molecular dynamic simulation of salty water icing

Although the TIP4P/Ice[7] and TIP4P/2005[8,9] models have good applications in the MD simulation of water freezing, the two atomic models require large amounts of computational resources even with the solid-liquid coexistence technique, and simulating the freezing process of high salinity water requires more computational resources due to the freezing suppression by the saline ions. Consequently, the mW-ion model developed by Molinero et al.[10] with high accuracy and low computational cost is adopted. In the mW-ion model, the water model is mW (monatomic water, mW), and the ion stands for Na^+ and Cl^-. This model spends less than 1% of the computational cost of other atomic models[11], and can precisely describe the structure of water molecules in liquid water, ice crystal, and amorphous solid water, which has been widely used in the MD simulation of the freezing[11–13]. By comparing with experimental data, the mW-ion model can accurately describe the effect of ions on the structure of water, as well as the properties of NaCl solution structure, density, and diffusion coefficient. The Stillinger-Weber force field is used to describe the interactions between the water and the Na^+ and Cl^- in the mW-ion model[10,11]. Additionally, the Yukawa force field is used to describe the repulsive forces between Na^+-Na^+ and Cl^--Cl^-. The force field equations and detailed parameters of the mW-ion model can be found in literature[10].

MD simulations are implemented using the Large-scale Atomic/Molecular Massively Parallel Simulator (LAMMPS) published by Sandia Labs[14]. The method of coexistence of the NaCl solution and the hexagonal ice (*i.e.*, Ice Ih, acting as the seed to trigger the freezing of the NaCl solution) is applied. The initial configuration for the coexistence of the NaCl solution and the Ice Ih was prepared as follows: (i) the Ice Ih consisting of 1152 mW water molecules is constructed using the GenIce tool, and then it is relaxed for 50 ns at 261 K and atmospheric pressure (anisotropic controlled pressure) under the NPT ensemble to avoid the effect of internal stresses in Ice Ih. The equilibrated structure of the Ice Ih is shown in Figure S6a with the dimensions of 45.90 Å × 43.16 Å × 17.71 Å (X × Y × Z). (ii) The Ice Ih configuration obtained in step (i) (Figure S6a) is replicated three times along the Z-axis, and the structure is heated for 10 ns at 300 K to produce liquid water under NVT ensemble. Then 105 Na and Cl ion pairs are randomly inserted into the liquid water, followed by heating the system for 10 ns at 300 K to obtain a 9 wt% NaCl solution. Finally, the NaCl solution is relaxed for 50 ns under the NP_ZT ensemble at 261 K and atmospheric pressure, and only the pressure perpendicular to the solid-liquid interface (the Z-axis direction) is controlled. The equilibrated structure is shown in Figure S6b with the dimensions of 45.90

Å × 43.16 Å × 53.69 Å (X × Y × Z). (iii) The initial model of solid-liquid coexistence as shown in Figure S6c with dimensions of 45.90 Å × 43.16 Å × 163.71 Å (X × Y × Z) is formed by putting the Ice Ih obtained in step (i) (Figure S6a) and the NaCl solution obtained in step (ii) (Figure S6b) in contact in the Z-axis direction.

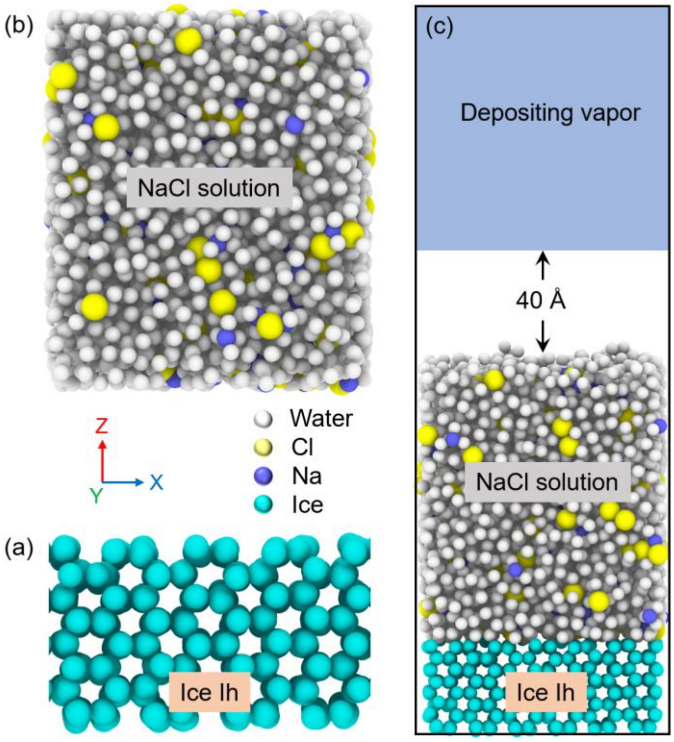

FIGURE S6. Schematic of the initial model configuration in the MD simulation of salty water icing. (a) Hexagonal ice Ih, (b) NaCl solution, and (c) initial coexistent configuration of the NaCl solution with Ice Ih. A depositing vapor region is set above the NaCl solution region and there is a certain distance between these two regions.

The initial model is divided into three regions: the Ice Ih region (containing 1152 mW water molecules), the middle NaCl solution region (containing 3456 mW water molecules, 105 Na and Cl ion pairs), and the top depositing vapor region where water vapor molecules are generated. The distance between the upper boundary of the NaCl solution region and the lower boundary of the water vapor region is 40 Å. The surface of the ice slab in contact with the solution is the secondary prismatic with the fastest freezing rate of Ice Ih. It should be emphasized that, for the solid-liquid coexistence method, it is strictly correct to control the pressure only in the direction perpendicular to the solid-liquid interface. After the initial configuration is prepared, the freezing of the NaCl solution is performed. Firstly, the

prepared initial configuration under NP_ZT ensemble at 261 K and atmospheric pressure (only the pressure in the Z-axis direction is controlled) is simulated for 400 ns, and the simulation results are shown in Figures 1D, E and F. Then, a water molecule with a velocity of 100 m/s in the negative direction of the Z-axis is generated every 0.05 ns at a random position of the depositing vapor region, and 100 water molecules are generated in 5 ns. After that, the generation of water molecules is stopped to freeze for 35 ns. The above procedure is repeated 10 times for a duration of 400 ns, and the simulation results are shown in Figures 4D and E.

To quantitatively study the freezing process of salty water, it is necessary to distinguish the structure and number of ice-like and water-like molecules and thus determine the solid-liquid interface. Therefore, the CHILL+ algorithm is used to identify the structure of a water molecule, which can effectively identify cubic ice, hexagonal ice, interfacial ice, hydrates, and interfacial hydrates[15]. In this work, the cubic ice, hexagonal ice, and interfacial ice are classified as ice-like structure (denoted as ice in Figure S6), while the rests are water-like structure (denoted as water in Figure S6). As shown in Figure S7, to identify the solid-liquid interface, the coexistence system of the Ice Ih and the NaCl solution is firstly identified by the CHILL+ algorithm, and then water molecules belonging to the ice-like structure are obtained, at which the location of the solid-liquid interface can be roughly obtained. However, the ice-water interface is not flat. Therefore, the Gibbs interface identifying method is used to locate the solid-liquid interface more precisely[16]. The process of this method is as follows: the ice-like system is divided into 12 × 72 (X × Z) bins in the X-Z plane, the density of ice-like molecules in each bin is calculated, and then the solid-liquid interface position is obtained by fitting the following equation[16]:

$$\rho(z) = \frac{\rho_{ice}}{2} - \frac{\rho_{ice}}{2}\tanh\left(\frac{2(z-z_{SI})}{d_{SI}}\right) \quad \text{(S1)}$$

where $\rho(z)$ is the density of the ice-like molecules in each bin along the Z-axis, ρ_{ice} is the mean density of the ice-like system (0.59 amu/Å3, amu is the relative atomic mass), z_{SI} is the position of the solid-liquid interface in the z-axis direction, d_{SI} is the thickness of the solid-liquid interface. Since the ice-like system is divided into 12 blocks in the X-axis direction, Eq. (S1) needs to fit 12 times to obtain 12 positions of the solid-liquid interface. Finally, the average value of these 12 values is taken as the position of the solid-liquid interface.

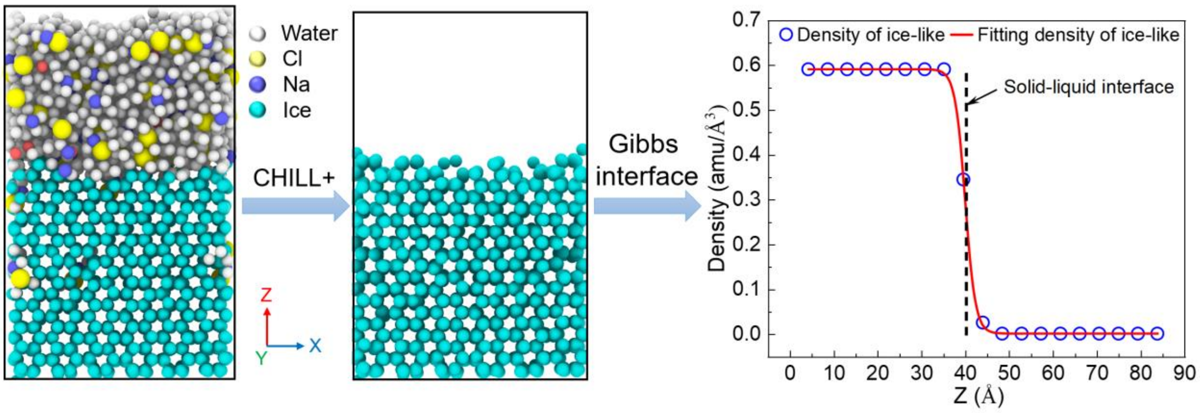

FIGURE S7. Schematic diagram of the identification process of the solid-liquid interface.

S7 Growth of dense ice crystals during the freezing stage of salty droplets

Because of the mussy growth of ice crystals in the freezing stage, one cannot see the regular ice front propagation in a 3D salty droplet (Figure 1B). To clearly observe the growth of dense ice crystals in the freezing stage of salty droplets, we design a Hele-Shaw cell (Figure S2b) and conduct icing experiments of salty droplet slices on experimental surfaces. Figure S8 shows time-lapsed 2D images of the icing process of a salty droplet slice. As seen, the growth of these dense ice crystals is clear in 2D images.

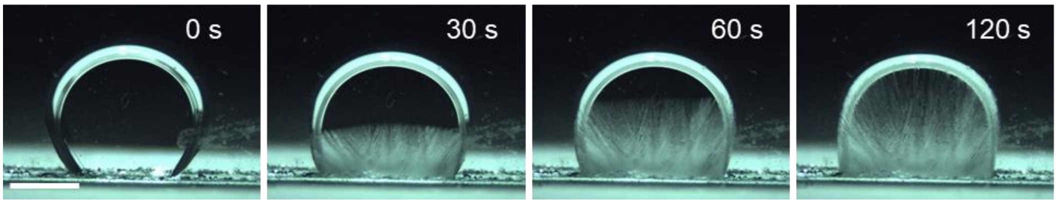

FIGURE S8. Icing process of a salty droplet slice in the Hele-Shaw cell (ω=6% and T_w= −15℃). The front of the ice crystals is nearly smooth. The scale bar is 5 mm.

From Figure S8, although the ice crystal growth is discontinuous that the gaps between ice crystals are filled with concentrated brine water, there is a most possible path to allow one aggregation of ice crystals to grow the longest, which is from the solid substrate to the droplet top. We show this using a schematic in Figure S9. As shown, it is the growth of the longest ice crystal which decides the freezing time, because after the longest ice crystal contacts the top of the droplet, the ice crystals can only be densified inside the droplet, extruding the brine film at the top of the droplet, which is defined as the end of freezing. In addition, in the path for the longest ice crystal, the ice crystal growth is assumed not to be disturbed by other ice crystals. Hence, the heat transfer in this ice crystal can be regarded as the one-dimensional Stefan problem[17] and this is the basis for the established model to predict the freezing time of salty droplets.

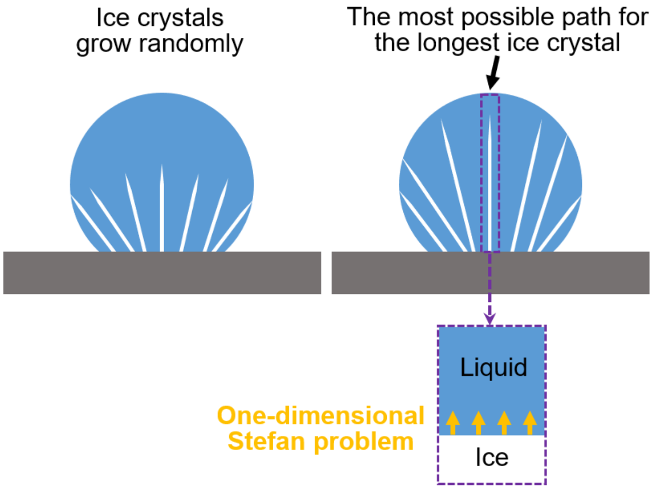

FIGURE S9. Schematic depicting the basis of the established model to predict the freezing time of salty droplets. Ice crystals grow randomly and unevenly inside the salty droplets. There is a most possible path (marked by the dashed box) to allow an ice crystal to grow the longest, which decides the freezing time. The single ice crystal growth in its growth path can be regarded as one-dimensional Stefan problem.

S8 Semi-empirical model to predict the freezing time of salty droplets

For water droplet icing, the freezing stage constitutes a classic Stefan problem, the energy conservation at the freezing front can be described by:

$$q_{ice} - q_{mix} = \rho_{ice} L_{mix} \frac{dH}{dt} \qquad \text{(S2)}$$

where q_{ice}, q_{mix} is the heat flux of ice and mixture, ρ_{ice} is the density of ice, L_{mix} is the latent heat of solidification, H is the freezing front height, and t is time. Since the water-ice mixture remains at a constant temperature of 0°C (freezing point), q_{mix}=0 in Eq. (S2). Under our experimental conditions that the droplet radius r is ~ 1 mm and the supercooling ΔT is ~ 15°C, given that L_{mix} =3.35×10^5 J/kg, ρ_{ice}=920 kg/m^3, k_{ice} =2.4 W/m·K, $C_{p,ice}$ = 2100 J/kg·K, and D_{ice} =1.24×10^{-6} m^2/s, the Stefan number, St = $C_{p,ice}\Delta T/L_{mix}$, is less than 0.1, indicating the domination of latent heat released from the ice front. St also represents the ratio of the time scale of thermal diffusion in ice and the time scale of solidification, τ_d/τ_{sol}, *i.e.*, τ_d is much smaller than τ_{sol}. Since $\tau_d = r^2/D_{ice}$, is in the scale of ~ 0.8 s, τ_{sol} is estimated to be in the scale of ~ 10 s. This is consistent with the experiments that it takes ~ 10 s for a droplet to freeze (Figs. 1A-B). To sum up, under our experimental conditions, the time scale of thermal diffusion in the ice layer is much smaller than the time scale of solidification, the heat transfer in the ice layer can be approximated as a one-dimensional quasi-steady procedure, and the temperature distribution satisfies an approximately linear distribution. Therefore, Eq. (S2) can be described by Eq. (1) in the main text. Integration of Eq. (1) yields an expression about the freezing time (t_{fr}) of water droplet, *i.e.*, Eq. (2).

As for the salty droplet icing, concentrated brine exists in the crevices of the ice crystals, resulting in a discontinuous freezing front (Figure S8). However, specifically for the growth of each aggregate of ice crystals, as depicted in Figure S9, we can still regard the freezing of salty droplet as a Stefan problem. During the icing process of a salty droplet, the internal temperature of the droplet rapidly increases from a certain degree of supercooling to the recalescence point, and then slowly decreases until the freezing process is finished (Figure S3a). According to our measurements, the temperature of a salty droplet after the nucleation-recalescence stage (T_i) is very close to the freezing point temperature of the salty solution with the same salinity (Figure S10a). Regarding the droplet temperature just after the freezing stage (T_{fr}), our experiments suggest that T_{fr} is usually higher than the cold surface temperature T_w. To sum up, during the freezing process, the temperature of the salty droplet decreases from T_i to T_{fr}. Simultaneously, the concentrated brine inside the droplet continues to be concentrated from ω_i to ω_{fr}. Figure S10b shows

the schematic of the changes of droplet temperature and salinity from the onset to the end of the freezing stage in the phase diagram of NaCl solution.

In other words, the unfrozen liquid brine always keeps saturated in the freezing stage and its concentration (ω) increases as the droplet temperature (T) decreases. We assume that ω increases linearly with the decrease of T (Figure S10b) and the fitting formula between them is:

$$T = -0.87157\omega + 274.50 \quad \text{(S3)}$$

As mentioned above, the droplet temperature at the end of freezing T_{fr} is between T_i and T_w, so we assume T_{fr} as a function of T_i and T_w:

$$T_{\text{fr}} = a(T_i - T_w) + T_w \quad \text{(S4)}$$

where a is an empirical coefficient from experiments, which is a fixed value when ω is the same. Refer to the value of a in Table S1.

Since the internal temperature of droplet is decreasing in the freezing process, the temperature difference between the cold surface and the liquid brine inside the droplet ΔT is always changing. At first, $\Delta T = T_i - T_w$, and finally $\Delta T = T_{fr} - T_w$. For convenience, ΔT is directly averaged as:

$$\Delta T = \frac{T_i + T_{fr} - 2T_w}{2} \quad \text{(S5)}$$

Substituting Eq. (S5) into Eq. (2), we obtain the semi-empirical model for predicting the freezing time of salty droplets. The experimental data in Figures 2D and 2E validate the reliability of the model. It should be noted that, when ω=0 (*i.e.*, pure water droplet), $T_i = T_{fr} = 0$°C and $\Delta T = -T_w$, which is consistent with the prediction model for water droplet icing.

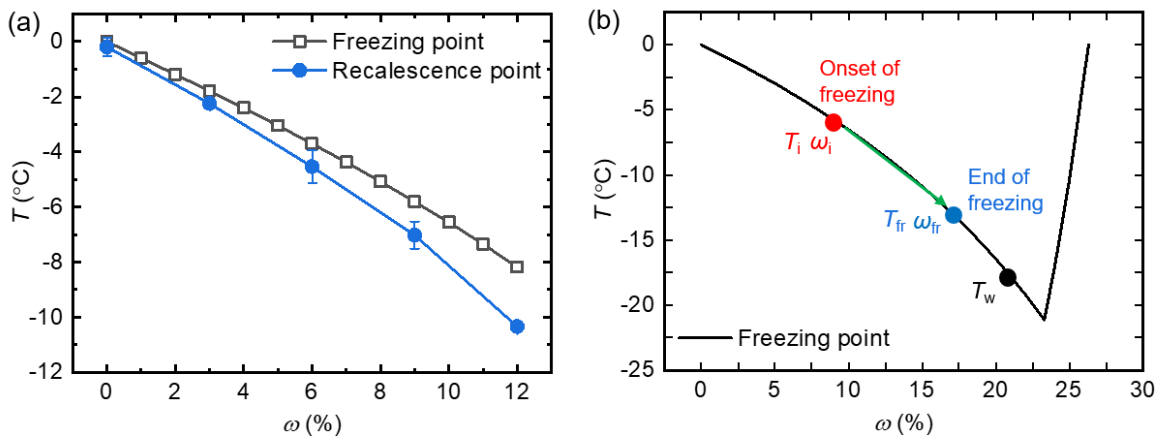

FIGURE S10. Temperature change of a freezing salty droplet. (a) Comparison of the recalescence point (T_i) and the freezing point at the same concentration (ω). Error bars of recalescence points display the standard deviations of parallel measurements. (b) Changes of droplet temperature and salinity from the onset to the end of the freezing stage in the phase diagram of NaCl solution.

TABLE S1. The value of coefficient (a) for different salt concentrations (ω).

ω (%)	a
3	0.27
6	0.20
9	0.22
12	0.37

S9 Estimation of the brine film thickness on top of freezing salty droplets

The observed brine film formation on top of salty droplets after freezing is a unique phenomenon that does not occur during icing of pure water droplets. To better describe this phenomenon, we measure the thickness of brine films on top of freezing salty droplets based on the optical images. We assume that after the freezing stage finishes, the solid contour of the freezing droplet is fixed by dense ice crystals. Then, due to the subsequent brine film formation, the contour line of the droplet will slightly rise. Therefore, we can roughly estimate the thickness of the brine film by measuring the rise distance of the droplet contour line.

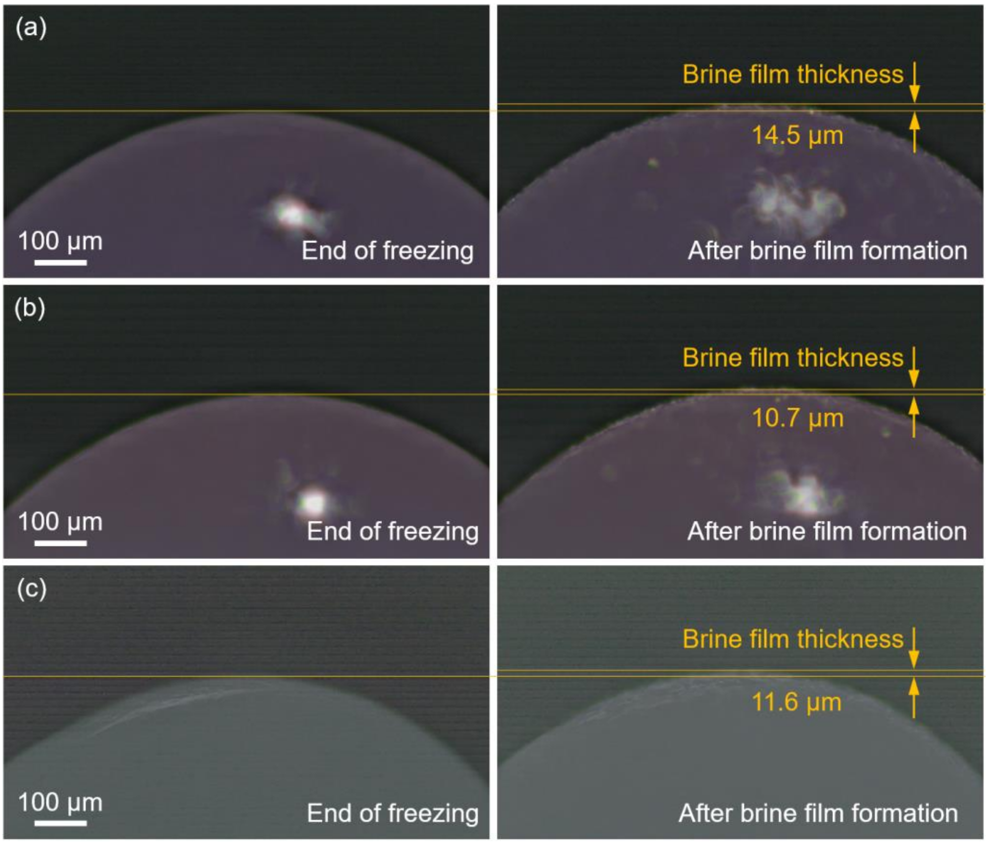

FIGURE S11. Thickness measurement of brine films on top of freezing salty droplets based on change of droplet contour. (a)-(c) show three examples of measurement. The distance between the two yellow lines in each measurement represents the elevation of the droplet contour line, which is roughly regarded as the thickness of the brine film. The experimental conditions for (a)-(c) are: droplet volume V = 8 μL, salt concentration ω = 9%, surface temperature T_w = -15°C, and the air humidity RH=30%.

Figure S11 show three measurement examples. During every measurement, we first capture the contour of the droplet at the end of freezing using a microscope, and at this time the droplet contour is smooth. After the brine film formation, we capture the contour of the droplet again. Notice that the droplet contour line after brine film formation will become unsmooth because of the ice sprouting inside the brine film. Then we measure the rise distance of the droplet contour line, as marked by the two yellow lines in each measurement in Figure S11, and this rise distance is roughly regarded as the thickness of the brine film. The measurement results show that, the thicknesses of brine films are all in the scale of ~ 10 μm.

S10 Analogy experiments for the measurement of ice precipitation rate and condensation rate

We design an analogy experiment to simulate the ice sprouting phenomenon at top of a frozen salty droplet. As seen in Figure S12, compared with the experimental setup for droplet icing, the setup of the analogy experiments has a copper sink instead of a solid surface. In the experiment, we put a silver iodide disc at the bottom of the copper sink and inject brine into the copper sink to just immerse the silver iodide disc. The copper sink is cooled to a temperature that corresponds to the freezing point temperature of the brine by the semiconductor cooler. Since copper has good thermal conductivity and the brine film in the copper sink is very thin, we assume that both the liquid film and the silver iodide disc quickly reach thermal equilibrium with the copper sink, *i.e.*, the temperature of the three is equal. In Figure S12, the blue-gray background area means a closed chamber and the humidity of the chamber can be tuned by the humidifier. The experimental conditions are listed in Table S2, where T_{air} and RH are the temperature and humidity of the air in the chamber, and T_f represents the cooper sink or brine temperature. It should be noted that the air humidity in the chamber has an uncertainty of ±5%.

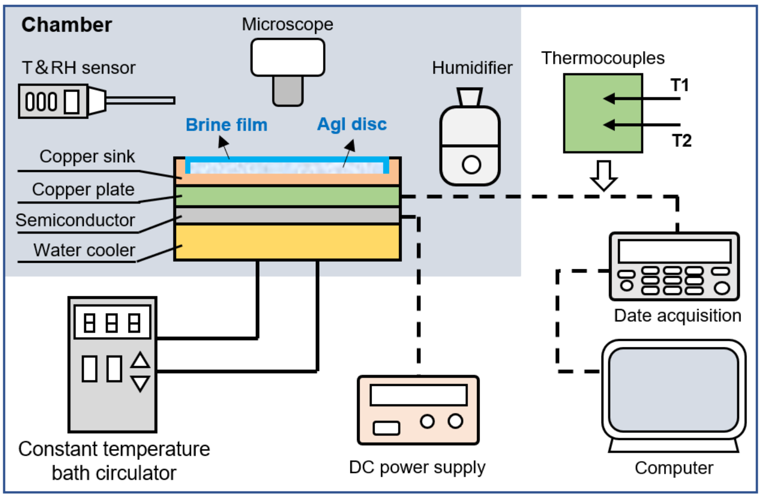

FIGURE S12. Setup of the analogy experiment. It is roughly the same as the experimental setup for droplet icing. Except, the experimental surface is replaced by a copper sink, in which brine water is injected and a silver iodide disc is immersed into the bine film.

TABLE S2. Experimental conditions.

T_{air} (°C)	T_f (°C)	ω (%)	RH (%)
26	-12	16	65
			80
			95

Figure S13 shows more details about how to measure the ice precipitation rate and the condensation rate in the analogy experiment. We first put a silver iodide disc in the copper sink as the substrate for the precipitation of ice crystals[18], and before that we measure the mass of the silver iodide disc (m_{AgI}). We then inject brine water into the copper sink and weigh the total mass of the silver iodide disc and brine ($m_{\mathrm{AgI,sol}}$). Thereafter, we cool the cooper sink and start the experiment in the closed chamber. Six hours later, we measure the total mass of the silver iodide disc and ice ($m_{\mathrm{AgI,ice}}$), as well as the total mass of the silver iodide disc, ice and the remaining liquid (m_{sum}). Considering that some ice crystals grow from the edge of the silver iodide disc and extend into the liquid, which are very brittle and easy to shed, to minimize the mass loss caused by ice crystal shedding, we use a filter screen (about 60-mesh) to quickly remove the silver iodide disc and ice from the copper sink, and put them into a prepared beaker for weighing. The weight is recorded as $m_{\mathrm{AgI,ice}}$. Then, we pour the remaining liquid in the copper sink into the beaker and weigh the mass m_{sum}. According to these data, the mass of precipitated ice can be calculated by $m_{\mathrm{AgI,ice}} - m_{\mathrm{AgI}}$, and the mass of condensed water vapor can be calculated by $m_{\mathrm{sum}} - m_{\mathrm{AgI,sol}}$. After dividing by the time, we can get the ice precipitation rate and the condensation rate.

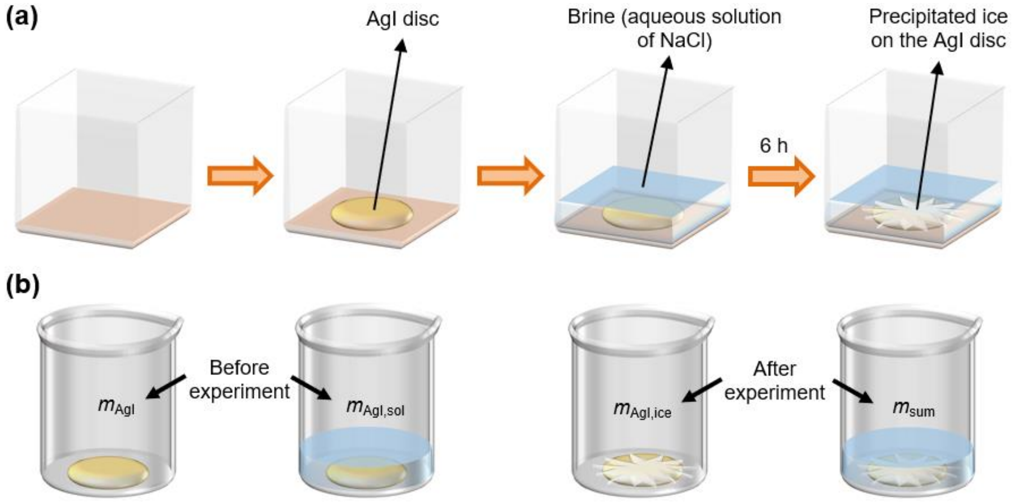

FIGURE S13. Schematic of the analogy experiments for the measurements of the ice precipitation rate and the condensation rate. (a) Procedure of the analogy experiment. (b) Mass measurements of precipitated ice and condensed water vapor. The mass of precipitated ice is equal to $m_{\mathrm{AgI,ice}} - m_{\mathrm{AgI}}$, where $m_{\mathrm{AgI,ice}}$ is the total mass of the silver iodide disc and ice after experiment and m_{AgI} is the mass of silver iodide disc before experiment. The mass of condensed water vapor is equal to $m_{\mathrm{sum}} - m_{\mathrm{AgI,sol}}$, where m_{sum} is the total mass of the silver iodide disc, ice and the remaining liquid after experiment and $m_{\mathrm{AgI,sol}}$ is the total mass of the silver iodide disc and brine before experiment.

S11 Condensation phase change driving force

According to the thermodynamic principles, the condensation phase change driving force is equal to the Gibbs free energy decrease (-Δg) when water molecule transfers from gas state to liquid state[19,20], which can be described by Eq. (3). Since the water vapor partial pressure can be expressed as a function of air temperature and humidity, Eq. (3) is described as:

$$-\Delta g = k_{\mathrm{B}} T_{\mathrm{f}} ln \frac{p_{v,s}(T_{\mathrm{air}})RH}{p_{v,s}(T_{\mathrm{f}})} \tag{S6}$$

where $p_{v,\mathrm{s}}(T)$ describes the water vapor saturation pressure at a temperature, which can be obtained from Table S3 when T is from 259 to 303 K. The function of $p_{v,\mathrm{s}}(T)$ can also be fitted as:

$$p_{v,s}(T) = e^{\frac{T-281.04}{14.04}} \tag{S7}$$

Therefore, the condensation phase change driving force can be estimated by the combination of Eq. (S6) and Eq. (S7) when T_{air}, RH and T_{f} are given. Here, T_{air} and RH are the temperature and humidity of the air, and T_{f} is the cooper sink or brine temperature. The condensation rate on the brine film surface should be proportional to the condensation phase change driving force. Figure 4C in the main text compares the variation trends of the condensation driving force, condensation rate and ice precipitation rate with relative humidity. It can be found that the variation trends of the three are in good agreement.

TABLE S3. The water vapor saturation pressure at different temperatures (259-303K).

T (K)	p_{sat} (kPa)	T (K)	p_{sat} (kPa)
259	0.181	283	1.227
261	0.217	285	1.401
263	0.259	287	1.597
265	0.309	289	1.817
267	0.368	291	2.062
269	0.437	293	2.337
271	0.517	295	2.642
273	0.611	297	2.982
275	0.705	299	3.36
277	0.813	301	3.778
279	0.935	303	4.241
281	1.072		

S12 Effects of air humidity and surface temperature on the ice sprouting phenomenon

To demonstrate the effect of experimental conditions on the discovered ice sprouting phenomenon, we have conducted experiments under a wider set of conditions of air humidity and surface temperature (i.e., supercooling). Figure S14 shows the ice sprouting phenomena inside brine films under various air humidity conditions (other conditions are the same) from both side and top view. As seen, taking the nucleation time of droplet freezing as zero time, for the air humidity *RH*=30% (ω=9%, *V*=4 μL, T_w=-15°C, and T_{air} =25°C), when t = 120 s, the sprouting ice crystals are not many. For the air humidity *RH*=60%, at t = 120 s, the sprouting ice crystals are much denser. For the air humidity *RH*=90%, even at t = 90 s, the sprouting of ice crystals is already evident. In other words, the higher the humidity in the air, the more obvious the ice sprouting phenomenon.

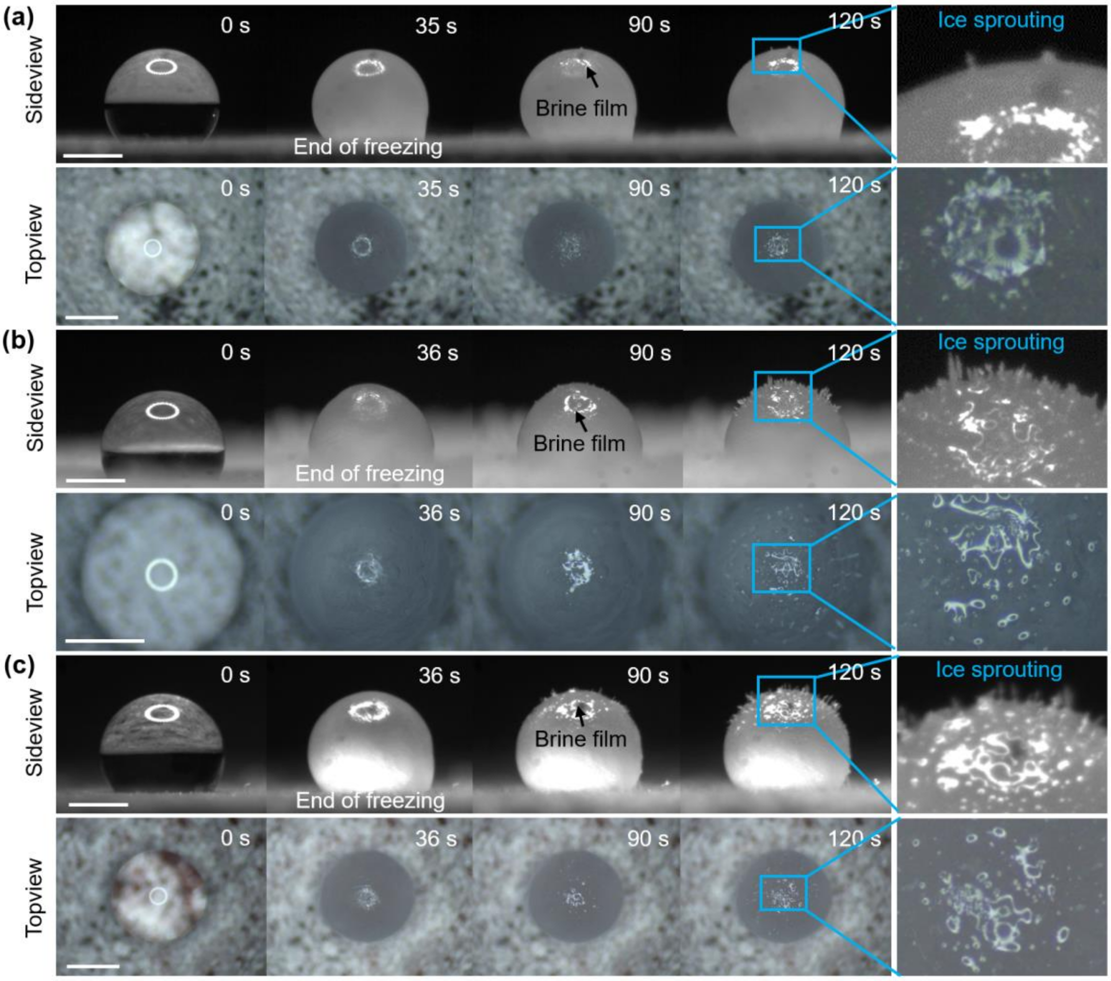

FIGURE S14. Ice sprouting in the brine film under various air humidity conditions. (a) *RH*=30%, (b) *RH*=60%, and (c) *RH*=90%. All experiments are conducted under ω=9%, *V*=4 μL, T_w=−15°C, and T_{air} =25°C. All scale bars indicate 1 mm.

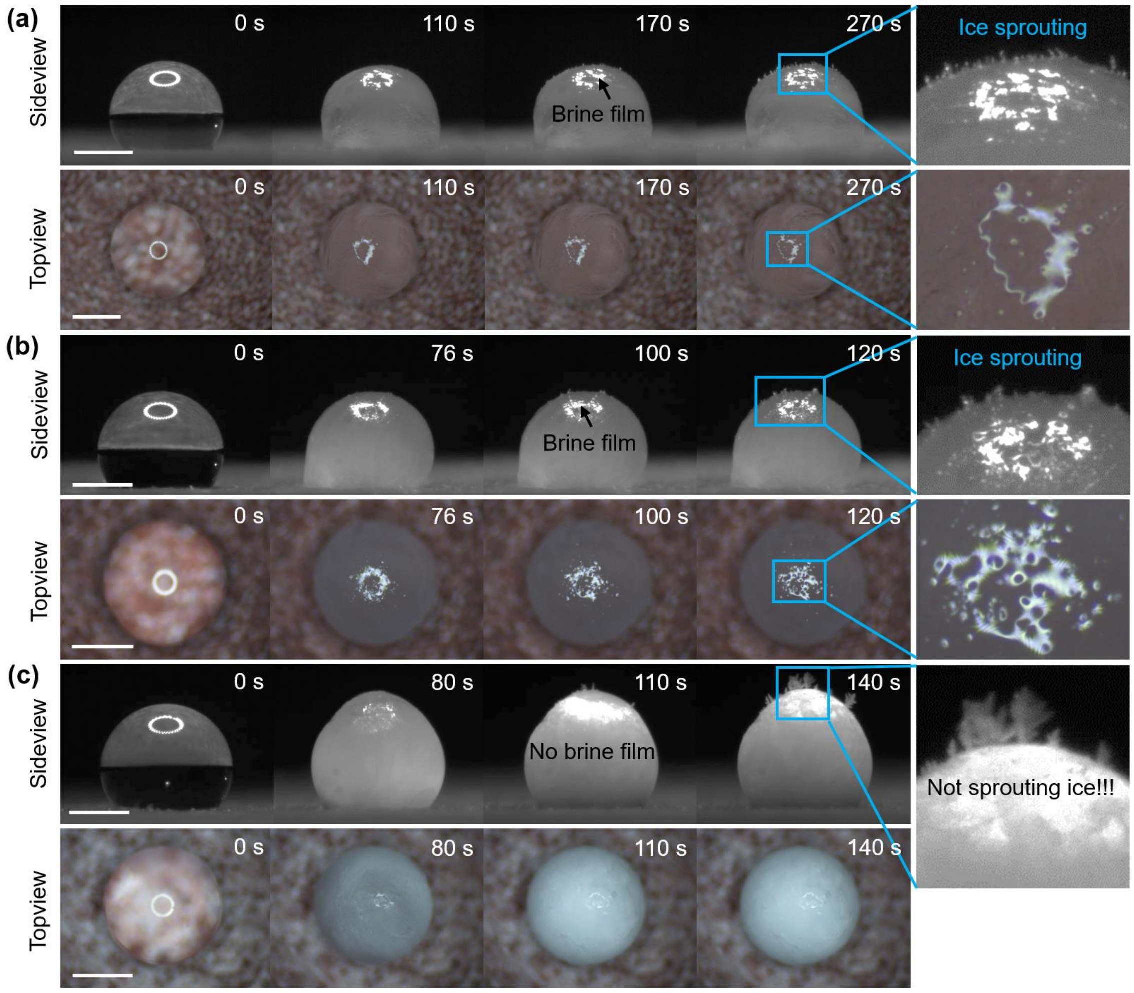

FIGURE S15. Ice sprouting in the brine film under various surface temperatures. (a) T_w=−10°C, (b) T_w=−15°C, and (c) T_w=−30°C. All experiments are conducted under ω=9%, V=4 μL, T_{air}=25°C, and RH=40%. It should be noted that when T_w=−30°C, no brine film forms and the ice crystals on top of the droplet are not sprouting ice. All scale bars indicate 1 mm.

Figure S15 shows the ice sprouting phenomena inside brine films under various surface temperatures (other conditions are the same). As shown in Figure S15a, the ice sprouting phenomenon occurs when the surface temperature T_w=−10°C (ω=9%, V=4 μL, T_{air} =25°C, and RH=40%). Under this condition, the supercooling for the salty droplet is less than 5°C, indicating that the ice sprouting phenomenon can occur under small supercooling. It should be noted that, if the supercooling is too small (such as near 0°C), it is difficult for droplets to freeze, let alone the ice sprouting phenomenon. However, when the supercooling is too strong that the surface temperature is lower than −21.1°C (the eutectic temperature of NaCl solution), the ice sprouting phenomenon will not occur. Figure S15c shows this

case. Because the temperature is lower than the eutectic temperature, the salty droplet finally becomes a complete solid phase with no brine film on its top. Therefore, the ice sprouting cannot exist. Notice that the ice crystals on top of the droplet are frost crystals due to the desublimation effect (Figure S15c), instead of sprouting ice crystals.

S13 Effect of salt ions on the ice sprouting phenomenon

To broaden the universality of the ice sprouting phenomenon, we have conducted droplet icing experiments using other salt solutions, including $MgCl_2$, $CaCl_2$, and KCl solutions. The results in Figure S16 show that, for salty droplets of $MgCl_2$, $CaCl_2$, and KCl solutions, the brine film formation and the inside ice sprouting on top of freezing droplets can also occur, indicating the universality of these phenomena for different salt ions.

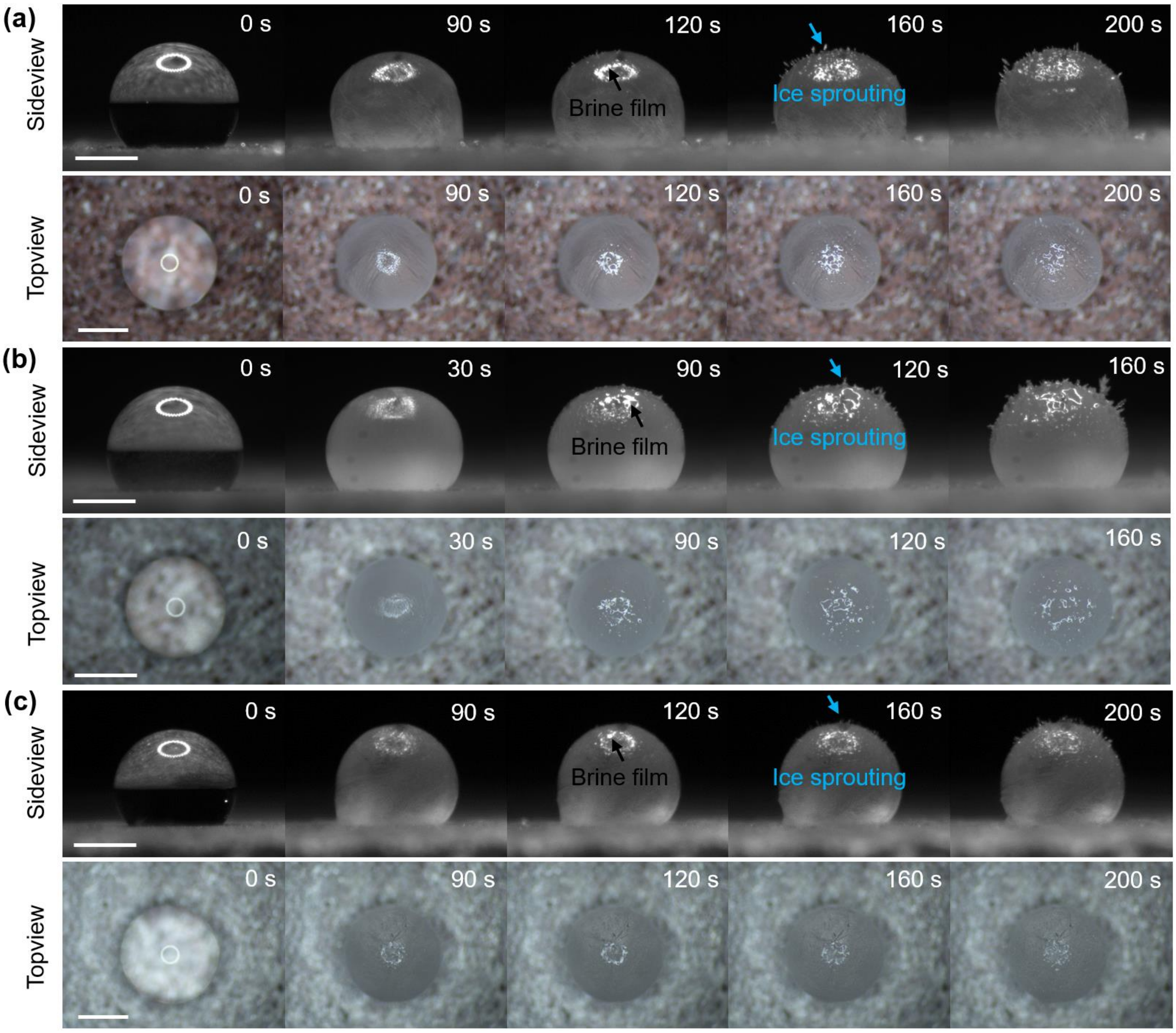

FIGURE S16. Ice sprouting in the brine film for different salt ions. (a) $MgCl_2$, ω=10%, and T_w=−15°C; (b) $CaCl_2$, ω=20%, and T_w=−24°C; (c) KCl, ω=6%, and T_w=−10°C. All experiments are conducted under conditions of V=4 μL, T_{air}=25°C, and RH=60%. Due to the different eutectic points of different salt solutions, we need to choose different concentrations and surface temperatures to make the droplets freeze. All scale bars indicate 1 mm.

S14 Salty droplet icing on hydrophilic surfaces

Figure S17 shows icing process of a salty droplet on hydrophilic bare aluminum surfaces (contact angle is ~80°) from both side view and top view. The icing conditions are V= 4 μL, ω=9%, T_w= −15°C and RH=50%. As seen, the nucleation/recalescence, freezing, brine film formation, and ice sprouting in the brine film are all consistent with those on hydrophobic surfaces shown in the main text, indicating that the reported results are general and not affected by the properties of the solid surface or the shape of the droplets.

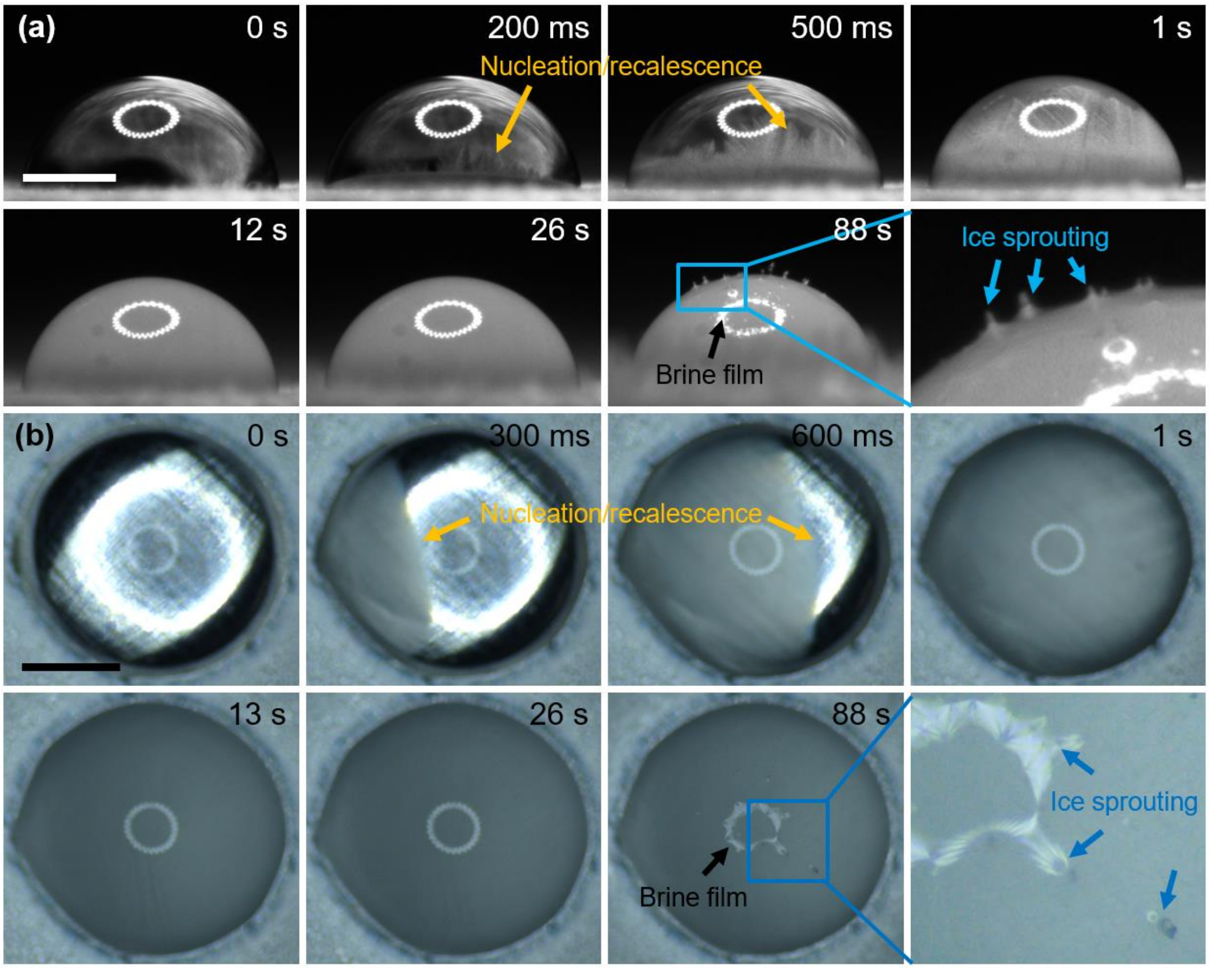

FIGURE S17. Icing process of a salty droplet on hydrophilic bare aluminum surfaces (V= 4 μL, ω=9%, T_w= −15°C and RH=50%). (a) Side views; (b) Top views. All results resemble those on hydrophobic surfaces. All scale bars indicate 1 mm.

Supplementary References

1. Chu, F., Gao, S., Zhang, X., Wu, X. & Wen, D. Droplet re-icing characteristics on a superhydrophobic surface. Appl. Phys. Lett. 115, 073703 (2019).
2. Tavakoli, F. & Kavehpour, H. P. Cold-induced spreading of water drops on hydrophobic surfaces. Langmuir 31, 2120 (2015).
3. Lambley, H. et al. Freezing-induced wetting transitions on superhydrophobic surfaces. Nat. Phys. (2023). DOI: 10.1038/s41567-023-01946-3.
4. Marin, A. G., Enriquez, O. R., Brunet, P., Colinet, P. & Snoeijer, J. H. Universality of tip singularity formation in freezing water drops. Phys. Rev. Lett. 113, 054301 (2014).
5. Jung, S., Tiwari, M. K., Doan, N. V. & Poulikakos, D. Mechanism of supercooled droplet freezing on surfaces. Nat. Commun. 3, 615 (2012).
6. Schremb, M. & Tropea, C. Solidification of supercooled water in the vicinity of a solid wall. Phys. Rev. E. 94, 052804 (2016).
7. Abascal, J. L. F., Sanz, E., García Fernández, R. & Vega, C. A potential model for the study of ices and amorphous water: TIP4P/Ice. J. Chem. Phys. 122, 234511 (2005).
8. Luo, S. et al. Molecular understanding of ion rejection in the freezing of aqueous solutions. Phys. Chem. Chem. Phys. 23, 13292 (2021).
9. Conde, M.M., Rovere, M. & Gallo, P. Spontaneous NaCl-doped ice at seawater conditions: focus on the mechanisms of ion inclusion. Phys. Chem. Chem. Phys. 19, 9566 (2017).
10. DeMille, R. C. & Molinero, V. Coarse-grained ions without charges: reproducing the solvation structure of NaCl in water using short-ranged potentials. J. Chem. Phys. 131, 034107 (2009).
11. Moore, E. B. & Molinero, V. Structural transformation in supercooled water controls the crystallization rate of ice. Nature 479, 506 (2011).
12. Hussain, S. & Haji-Akbari, A. Role of nanoscale interfacial proximity in contact freezing in water. J. Am. Chem. Soc. 143, 2272 (2021).
13. Lupi, L. et al. Role of stacking disorder in ice nucleation. Nature 551, 218 (2017).
14. Plimpton, S. Fast parallel algorithms for short-range molecular dynamics. J. Comput. Phys. 117, 1 (1995).
15. Nguyen, A. H. & Molinero, V. Identification of Clathrate Hydrates, Hexagonal Ice, Cubic Ice, and Liquid Water in Simulations: the CHILL+ Algorithm. J. Phys. Chem. B. 119, 9369 (2015).
16. de Ruijter, M. J., Blake, T. D. & Coninck, J. D. Dynamic wetting studied by molecular modeling simulations of droplet spreading. Langmuir 15, 7836 (1999).
17. Pinus, V. K. & Taylor, P. L. Stability and instability in crystal growth: Symmetric solutions of the Stefan problem. Phys. Rev. B 32, 5362 (1985).
18. Vonnegut, B. The Nucleation of Ice Formation by Silver Iodide. J. Appl. Phys. 18, 593 (1947).
19. Gránásy, L. Diffuse interface theory for homogeneous vapor condensation. J. Chem. Phys. 104, 5188 (1996).
20. Pellenq, R. J., Coasne, B., Denoyel, R. O. & Coussy, O. Simple phenomenological model for phase transitions in confined geometry. 2. Capillary condensation/evaporation in cylindrical mesopores. Langmuir 25, 1393 (2009).
