# Supplementary material for: Interfacial ice sprouting during salty water droplet freezing
Source: Nat Commun. 2024 Mar 13;15:2249. doi: 10.1038/s41467-024-46518-y (PMC10937636; doi:10.1038/s41467-024-46518-y)
Supplement: Supplementary file 3 — Description of Additional Supplementary Files [file 41467_2024_46518_MOESM3_ESM.docx]

**Description of Additional Supplementary Files**

**File Name: Supplementary Video 1
Description:** Comparison of water droplet icing and salty droplet icing.

**File Name: Supplementary Video 2
Description:** The ice sprouting phenomenon during salty droplet icing.

**File Name: Supplementary Video 3
Description:** Ice precipitation on the silver iodide disc in the analogy experiment.

**File Name: Supplementary Video 4
Description:** Molecular dynamic simulation of the whole process of salty water icing.
